# Ferroptosis‐related gene CHAC1 is a valid indicator for the poor prognosis of kidney renal clear cell carcinoma

**DOI:** 10.1111/jcmm.16458

**Published:** 2021-03-16

**Authors:** Deng Li, Shiwei Liu, Jie Xu, Lei Chen, Chaoliang Xu, Feiteng Chen, Zijie Xu, Yu Zhang, Shujie Xia, Yi Shao, Yi Wang

**Affiliations:** ^1^ Department of Urology Shanghai General Hospital Shanghai Jiao Tong University School of Medicine Shanghai China; ^2^ Nursing Department Wujiang Fifth People's Hospital Suzhou China; ^3^ Department of Urology Affiliated Hospital of Nantong University Nantong China

**Keywords:** CHAC1, ferroptosis, glutathione, kidney renal clear cell carcinoma, prognosis

## Abstract

To evaluate the validity of CHAC1 for predicting the prognosis of kidney renal clear cell carcinoma (KIRC) and to explore its therapeutic potential for KIRC, we conducted several bioinformatic analyses using the sequencing data and clinical information derived from online databases. We found CHAC1 is down‐regulated in KIRC samples when compared with normal samples but up‐regulated in KIRC samples with relatively higher malignancy and later stages. Univariate cox analysis and multivariate cox regression analysis were conducted and the results revealed up‐regulated CHAC1 is an independent risk factor for poor prognosis of KIRC. Further, the nomogram model based on the result of multivariate cox regression analysis was constructed and effectively predicted patients' 1‐year, 3‐year and 5‐year survival respectively. The correlation analyses showed CHAC1 is associated with the immune pathway markers of memory B cell, natural killer cell and type1 T helper cell as well as the checkpoint genes like ADORA2A, CD200, CD44, CD70, HHLA2, NRP1, PDCD1LG2 and TNFRSF18. Furthermore, experiments in vitro indicated CHAC1 could induce cell death in KIRC cell lines but had limited influence on cell migration and cell invasion. In conclusion, CHAC1 is found a valid indicator for poor prognosis of kidney renal clear cell carcinoma.

## 1 INTRODUCTION

Renal cell carcinoma (RCC) is the most common solid lesion in kidney and accounts for approximately 90% of kidney malignancies^1^ and 3% of all cancers.^2^ The EAU guideline indicated that the incidence of renal cell carcinoma is increasing yearly with an annual increase of about 2% during the last two decades.^2^ Among all histological subtypes of RCC, kidney renal clear cell carcinoma (KIRC) is the most common one and accounts for about 75% of all RCC.^2^ There is no doubt the investigation of KIRC is of great clinical importance.

Cancer cells are frequently found to be characterized with metabolic abnormalities. And reprogramming of metabolism is associated with tumour progression, the adaption to stress and anti‐tumour therapies.^3^, ^4^ For example, dysregulated glutathione (GSH) metabolism has been found playing a vital role in the initiation, progression and drug resistance of various kinds of malignant tumour.^5^ Glutathione is the most abundant thiol in living cells^6^ and acts as a reactive oxygen species (ROS) scavenger to prevent ROS damage from important cellular components.^7^ Previous researches have demonstrated that the depleting of GSH is associated with the accumulation of ROS and the induction of ferroptosis, a new form of iron‐dependent, programmed cell death,^8^ which has been found involved in the development of kidney diseases, especially in KIRC.^9^ For example, Heike Miess's research indicated that the glutathione redox system is essential to prevent ferroptosis in KIRC.^10^ Wu and his colleagues successfully constructed a new survival model for predicting the prognosis of KIRC using 32 ferroptosis‐related genes in his research.^11^ By bioinformatic analysis of KIRC sequencing data and clinical information derived from The Cancer Genome Atlas (TCGA) database, we identified the ferroptosis‐related gene ChaC Glutathione Specific Gamma‐Glutamylcyclotransferase 1 (CHAC1), which had also been mentioned in Wu's research, is significantly down‐regulated in KIRC samples when compared with normal samples. Given the known function of CHAC1 in GSH degradation and ferroptosis activation,^11^ we speculated that CHAC1 may play a role in the initiation or the progress of KIRC and the differential expression of CHAC1 might be a valid indicator for predicting the prognosis of KIRC.

To confirm our speculation, we conducted several bioinformatic analyses using the R scripts and website tools to investigate the clinicobiological function of CHAC1 in KIRC as well as the therapeutic potential of CHAC1 in KIRC. Furthermore, we performed experiments in vitro to demonstrate the function of CHAC1 in kidney cancer cell lines using overexpression vector of CHAC1. The results of current research would provide new strategies for predicting the prognosis of KIRC and explore the therapeutic potential of CHAC1 for KIRC.

## 2 MATERIALS AND METHODS

### 2.1 Data collection and processing

Pan‐cancer sequencing data and the RNA sequencing data of KIRC as well as its corresponding clinical information of 531 KIRC samples and 72 normal samples were downloaded from the TCGA database and processed using Bioconductor package in R statistical environment.^12^, ^13^ Differentially expressed genes were identified using the Bioconductor package of edgeR with criteria of |log_2_fold‐change(log_2_FC)|>2 and adjusted *P*‐value (adj.*P*) <.05. Predicted neoantigens for TCGA samples were obtained through The Cancer Immunome Atlas (TCIA).^14^ The tumour mutation burden (TMB) and microsatellite instability (MSI) were calculated as the total mutation incidences per million base pair and the number of insertion or deletion events that occurred in repeating sequences of genes respectively.^15^

### 2.2 Immune cell infiltration and tumour microenvironment analysis

The database‐derived website tool—Estimation of STromal and Immune cells in MAlignant Tumour tissues using Expression data (ESTIMATE)^16^ was used to evaluate the association between the components of tumour microenvironment and the expression of CHAC1 in KIRC. The database‐derived website tool—Tumor IMmune Estimation Resource (TIMER)^17^ was used to evaluate the association between immune cell infiltration (B cells, CD4+ T cells, CD8+ T cells, macrophages, neutrophils and dendritic cells) and the expression of CHAC1 in KIRC.

### 2.3 Genes set enrichment analysis (GESA)

Genes set enrichment analysis^18^, ^19^ was used to investigate the potential signaling pathways related to CHAC1 in KIRC. Based on expression of the CHAC1 in KIRC samples, we divided these samples into two groups of high expression group and low expression group. The predefined gene sets MSigDB (http://software.broadinstitute.org/gsea/msigdb/index.jsp) was used to conduct gene set enrichment analysis to study the significant status of various functional collections of groups with different expression of CHAC1 in KIRC.

### 2.4 Cell culture and samples

KIRC cell lines 786‐0 and CAKI‐1, renal tubular epithelial cell HK‐2 were stored in our laboratory. Cells were cultured according to the culture methods described in ATCC website respectively. Tissue samples of KIRC and pericarcinous tissues were collected from patients who received surgical treatment for KIRC in our institution. Extracted tissues were stored in −80°C or in 4% paraformaldehyde for further researches. The research was approved by the ethics committee of Shanghai General Hospital and all patients signed the informed consent.

### 2.5 Transfection

Overexpression vector of CHAC1 and its negative control (NC) vector were constructed by Gene Pharma. Vectors were transfected using lipo3000 reagent (Invitrogen) according to the manufacturer's protocol. Cells were incubated for 48 hours before further researches.

### 2.6 CCK‐8 assay

KIRC cell lines 786‐0 and CAKI‐1 transfected with overexpression vector of CHAC1 or its negative control vector were seeded into 96‐well plates with a density of 1000 cells/well. After culture for 24 hours, 10 μL CCK‐8 reagent was added to each well as scheduled. The optical density was measured after 2 hours incubation.

### 2.7 Cell migration and invasion assay

Transwell chambers (Corning Incorporated) with a pore size of 8 mm was used for cell migration and invasion assays. About 5 × 10^4^ 786‐0 and CAKI‐1 cells transfected with overexpression vector of CHAC1 or its negative control vector were seeded into the upper chamber. These cells were cultured with serum‐free medium. And medium containing 20% FBS was added to the lower chamber served as a chemoattractant. After incubation for 48 hours, 4% paraformaldehyde was used for fixing. Cells that migrated or invaded to the lower surface were stained in 10% crystal violet and counted under high‐power fields microscopically.

### 2.8 Quantitative real‐time PCR (qRT‐PCR)

Total RNA of cells and samples were extracted using TRIzol reagent (Thermo Fisher Scientific). RNA reverse transcription was performed using a PrimeScript™ RT reagent Kit with gDNA eraser (Takara) and quantitative real‐time PCR was performed using TBGreen^®^ Premix Ex Taq™ (Takara). The data were normalized using GAPDH levels and further analysed by the 2−ΔΔCT method.

### 2.9 Western blotting

Cells and samples were lysed using RIPA lysis buffer containing 1/100 PMSF (Roche). Total proteins were quantified using BCA protein assay kit (Pierce). Protein samples were resolved by 10% SDS‐PAGE gel and transferred to polyvinylidene difluoride membrane. And the stripes were incubated with primary antibodies against CHAC1 (Proteintech) and ACTB (abcam) at 4°C overnight, followed by incubating with a peroxidase‐conjugated goat anti‐rabbit IgG antibody (CST) for 2 hours at room temperature. Immunopositively bands were analysed using a FluorChem M system (ProteinSimple). The quantification of western blot images was conducted using Image J (Rawak Software Inc) and the targeted protein intensity was normalized with ACTB.

### 2.10 Immunohistochemistry

After antigen retrieval, samples were blocked in 10% BSA and incubated with primary rabbit antibodies against CHAC1 (Proteintech) for 30 minutes followed with incubation of biotinylated secondary antibodies (CST) for 30 minutes. Vectastain Elite ABC (Vector Laboratories) was added for 30 minutes and the reaction developed with 3,39‐diaminobenzidine DAB peroxidase substrate before counterstaining with hematoxylin. The expression of CHAC1 in KIRC samples and para‐carcinoma tissues was calculated using Image J (Rawak Software Inc).

### 2.11 Statistics

All statistical data analyses and figures were carried out using SPSS 25.0, GraphPad Prism 6.0 and R scripts/Bioconductor packages. Briefly, the Mann‐Whitney *U* test was used to compare the expression of CHAC1 between groups. The Kaplan‐Meier analysis was used to construct the survival curves of KIRC after dividing these patients into high and low risk according to the expression of CHAC1. Univariate cox analysis and multivariate cox regression analysis were performed to investigate whether CHAC1 can effectively predict the prognosis of KIRC. Moreover by the R “rms” package, we constructed a nomogram‐based model to predict patients' survival. The calibration curves and receiver operating characteristic curves (ROC) were conducted to verify the validity of the nomogram model. The spearman correlation test was used to assess the correlation between CHAC1 expression and targets of interest, such as neoantigens, MSI, TMB, mismatch repair (MMR) genes and methylation transferases. All statistical results with *P* < .05 were considered statistically significant.

## 3 RESULTS

### 3.1 The differential expression of CHAC1 in KIRC

**FIGURE 1:**
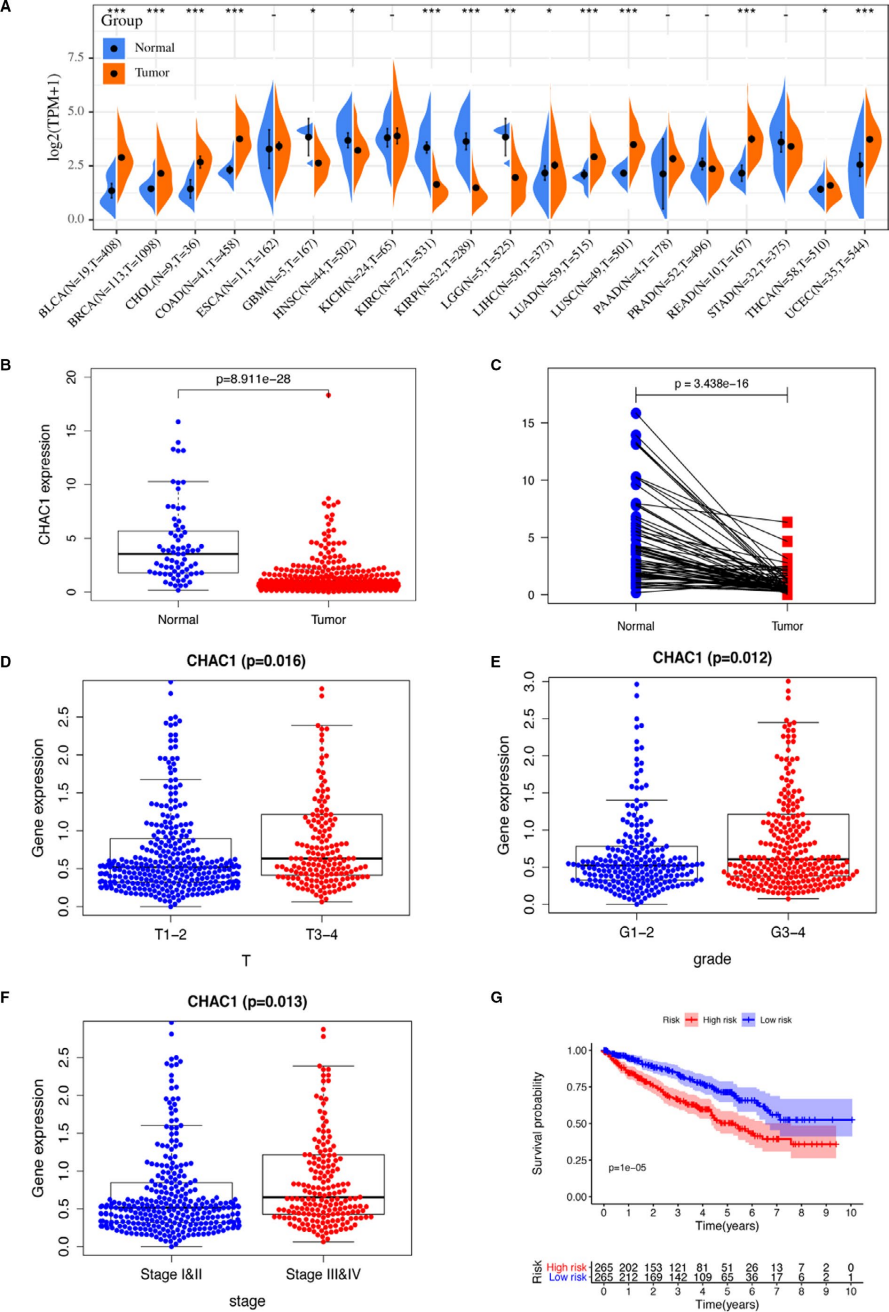
Differential expression of CHAC1 in KIRC. A, Pan‐cancer analysis of the expression of CHAC1. B, The expression of CHAC1 in KIRC samples and normal samples. C, The expression of CHAC1 in KIRC samples and their paired normal samples. D, The expression of CHAC1 in KIRC samples with different T stages. E, The expression of CHAC1 in KIRC samples with different grades. F, The expression of CHAC1 in KIRC samples with different total stages. G, The overall survival curve of KIRC. (*P < .05; **P < .01; ***P < .001)

We compared the expression of CHAC1 in different types of tumour samples and their corresponding normal samples using pan‐cancer sequencing data derived from TCGA database. We found CHAC1 is up‐regulated in bladder urothelial carcinoma (BLCA), breast invasive carcinoma (BRCA), cholangio carcinoma (CHOL), colon adenocarcinoma (COAD), liver hepatocellular carcinoma (LIHC), lung adenocarcinoma (LUAD), lung squamous cell carcinoma (LUSC), rectum adenocarcinoma (READ), thyroid carcinoma (THCA) and uterine corpus endometrial carcinoma (UCEC) and down‐regulated in head and neck squamous cell carcinoma (HNSC), KIRC, kidney renal papillary cell carcinoma (KIRP) and brain lower grade glioma (LGG) when compared with their corresponding normal samples (Figure 1A,B). We further compared the expression of CHAC1 in KIRC samples with their paired normal samples, and found CHAC1 is significantly down‐regulated in tumour tissues with the *P* value of 3.438e‐16 (Figure 1C). However, by investigating the expression of CHAC1 among KIRC samples with different grades and stages, we noticed CHAC1 is up‐regulated in KIRC samples with T3‐T4, G3‐4 and StageIII&IV when compared with the KIRC samples with T1‐2, G1‐2 and StageI&II respectively (Figure 1D‐F). And the survival curve also indicated up‐regulated CHAC1 is associated with higher mortality in KIRC (Figure 1G). Furthermore, we used external independent dataset from GEO database, ICGC database and ArrayExpress database to verify the prognostic effect of CHAC1. The results from GEO dataset of GSE6344 (N = 10, T = 10), GSE14994 (N = 11, T = 59) and the ICGC database (N = 45, T = 91) revealed CHAC1 is down‐regulated in KIRC samples when compared with normal samples with the *P*‐value of .029, 9.303e‐07, 1.744e‐10, respectively (Figure S1A‐C). The results from ArrayExpress:E‐MTAB‐1980 (T = 99) revealed the up‐regulation of CHAC1 is associated with relative short life span (*P* = .046) (Figure S1D).

### 3.2 Establish of predict model for the prognosis of KIRC

**FIGURE 2:**
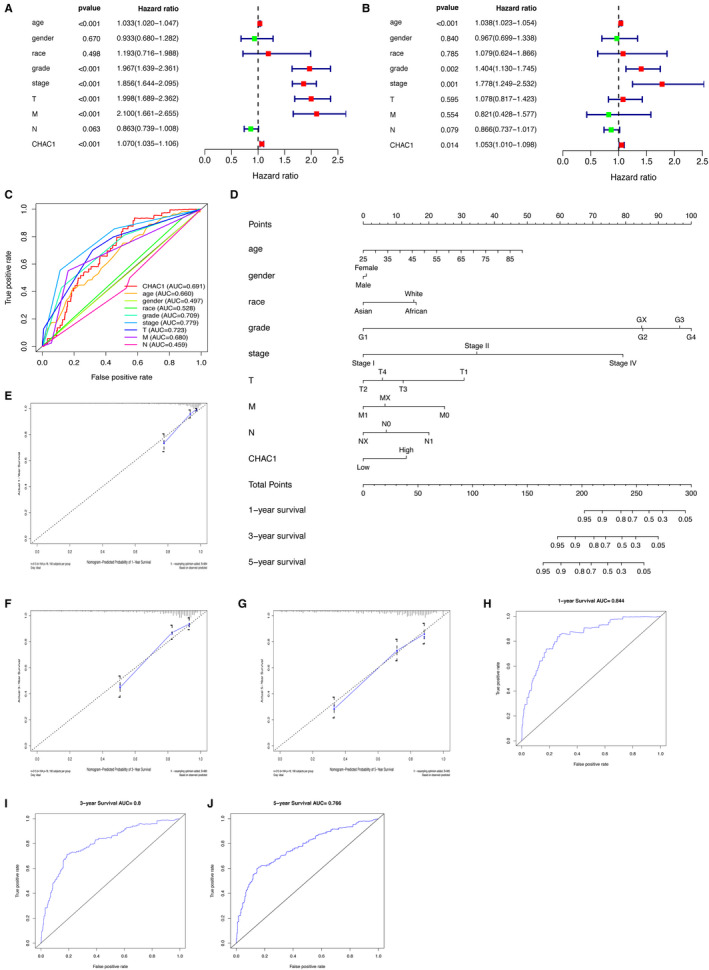
Establish of predict model for the prognosis of KIRC. A, Univariate cox analysis of the factors associated with overall survival of KIRC. B, Multivariate cox regression analysis of the factors associated with overall survival of KIRC. C, Multi‐ROC analysis of CHAC1 and the conventional prognostic factors. D, The nomogram model for predicting the prognosis of KIRC. E‐G, The calibration curves of the nomogram model for predicting patients' 1‐y survival, 3‐y survival and 5‐y survival. H‐J, The ROC curves of nomogram‐based model for predicting patients' 1‐y survival, 3‐y survival and 5‐y survival of KIRC

Univariate and multivariate cox regression analysis were used to identify the factors associated with overall survival (OS) of KIRC. The univariate cox analysis revealed that age (*P* < .001), grade (*P* < .001), stage (*P* < .001), T stage (*P* < .001), distant metastasis (*P* < .001) and the expression of CHAC1 (*P* < .001) are associated with OS (Figure 2A). Multivariate cox regression analysis revealed age (*P* < .001), grade (*P* = .002), stage (*P* = .001) and CHAC1 (*P* = .014) are associated with OS (Figure 2B). Multi‐ROC was used to compare the prognostic ability of CHAC1 with the conventional prognostic factors and the result revealed the area under the curve (AUC) of CHAC1 is 0.691 which is only less than the AUCs of grade (0.709), T‐stage (0.723) and total stage (0.779) (Figure 2C). A nomogram model including the above mentioned factors was constructed to predict the prognosis of KIRC (Figure 2D). The C‐index for the nomogram is 0.782. The calibration curves of the nomogram for the probability of 1‐year survival, 3‐year survival and 5‐year survival demonstrate good agreement between prediction and observation in the primary cohorts (Figure 2E‐G). The ROC curves revealed the nomogram‐based model could effectively predict patients' 1‐year survival, 3‐year survival and 5‐year survival with the AUCs of 0.844, 0.8 and 0.766 respectively (Figure 2H‐J).

### 3.3 Immunological features related to CHAC1 in KIRC

**FIGURE 3:**
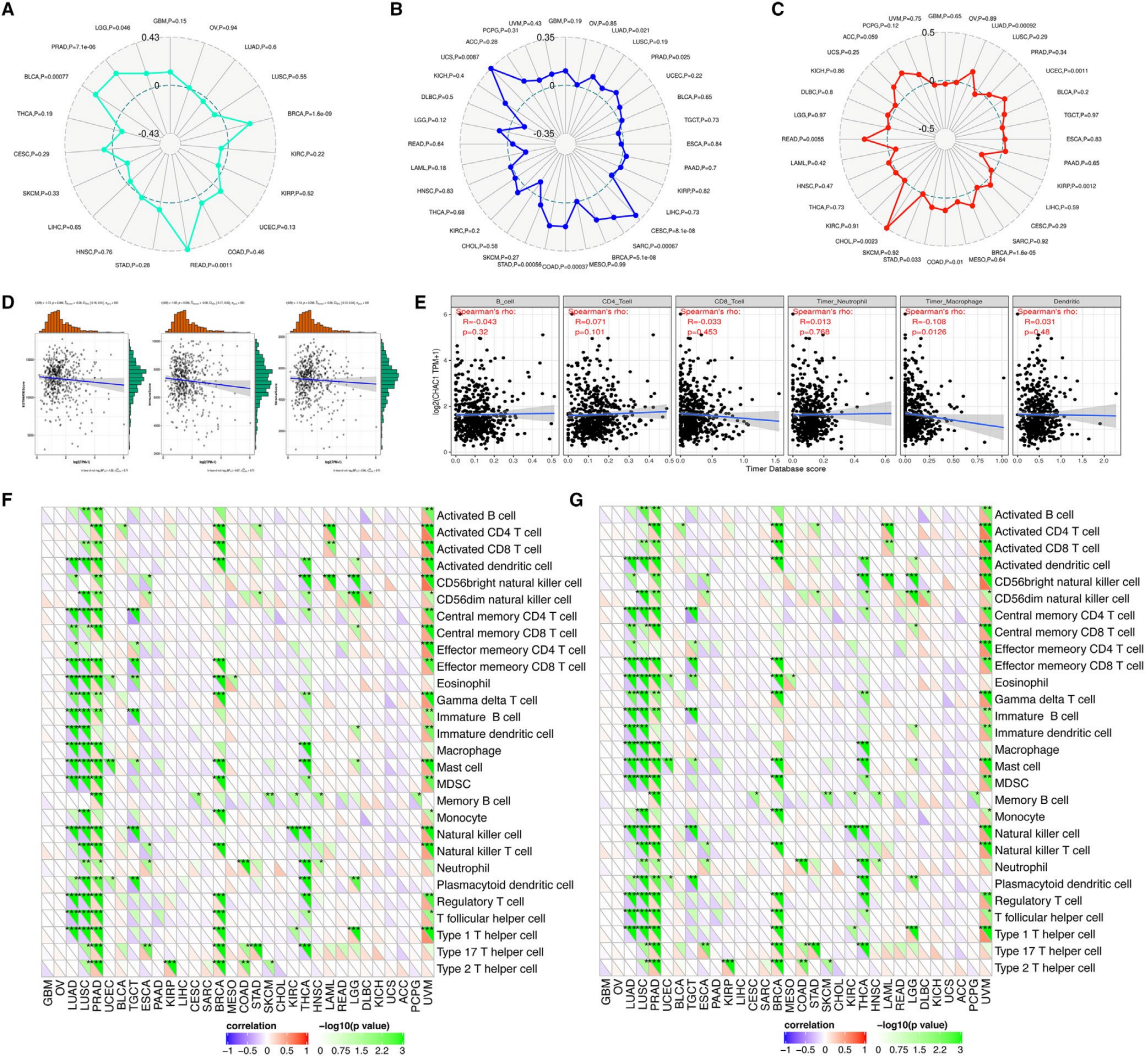
Immunological features related to CHAC1 in KIRC. A, The correlation analysis of CHAC1 expression and neoantigens. B, The correlation analysis of CHAC1 expression and MSI. C, The correlation analysis of CHAC1 expression and TMB. D, ESTIMATE: the correlation analysis of CHAC1 expression and tumour microenvironment. E, TIMER: the correlation analysis of CHAC1 expression and infiltration of immune cells. F, The correlation analysis of CHAC1 expression and acknowledged markers of immune pathway. G, The correlation analysis of CHAC1 expression and checkpoint genes. (*P < .05; **P < .01; ***P < .001)

Neoantigens, microsatellite instability and tumour mutation burden are valid biomarkers for immune therapy response in many kinds of tumour. Correlation analyses between CHAC1 and neoantigens, TMB and MSI revealed CHAC1 has limited influence on these biomarkers with the *P* value of .22, .2 and .91 respectively (Figure 3A‐C). However, significant association between CHAC1 and neoantigens were noticed in BLCA (*P* = .00077), prostate adenocarcinoma (PRAD) (*P* = 7.1e‐06), LGG (*P* = .046), BRCA (*P* = 1.6e‐09) and READ (0.0011) (Figure 3A). Moreover CHAC1 was also found correlated with MSI in uterine carcinosarcoma (UCS) (*P* = .0087), PRAD (*P* = .025), cervical squamous cell carcinoma and endocervical adenocarcinoma (CESC) (*P* = 8.1e‐08), sarcoma (SARC) (*P* = .00067), BRCA (*P* = 5.1e‐08), COAD (*P* = .00037) and stomach adenocarcinoma (STAD) (0.00056) (Figure 3B) and correlated with TMB in CHOL (*P* = .0023), READ (*P* = .0055), LUAD (*P* = .00092), UCEC (*P* = .0011), KIRP (*P* = .0012), BRCA (*P* = 1.6e‐05) and COAD (*P* = .01) (Figure 3C). Further, we used the website tools of TIMER and ESTIMATE to investigated the association between CHAC1 and immune cells infiltration as well as the construction of microenvironment in KIRC. The result from ESTIMATE revealed no statistical correlation was noticed between the expression of CHAC1 and the construction of microenvironment (Figure 3D). By the website tool of TIMER, except for macrophages (*P* = .0126), no clear association was noticed between CHAC1 and the infiltration of immune cells like B cells, CD4+ T cells, CD8+ T cells, neutrophils and dendritic cells (Figure 3E). To investigate the potential targets for the immunological therapy of KIRC, the mRNA sequencing data of KIRC was used to evaluate association between CHAC1 and the acknowledged markers of immune pathway as well as the checkpoint genes. The correlation analysis of CHAC1 and immune pathway markers revealed that CHAC1 influences the expression of markers of memory B cell (*P* < .05), natural killer cell (*P* < .001) and type1 T helper cell (*P* < .05) (Figure 3F). The correlation analysis of CHAC1 and the checkpoint genes revealed the expression of CHAC1 is associated with the expression of checkpoint genes like ADORA2A (*P* < .05), CD200 (*P* < .001), CD44 (*P* < .05), CD70 (*P* < .05), HHLA2 (*P* < .001), NRP1 (*P* < .01), PDCD1LG2 (*P* < .01) and TNFRSF18 (*P* < .01) (Figure 3G). Besides, we noticed the expression of CHAC1 is associated with various gene markers of immune cells as well as the checkpoint genes in LUAD, LUSC, PRAD, BRAC and especially, in uveal melanoma (UVM) (Figure 3F,G).

### 3.4 Correlation analyses of CHAC1 and MMR genes as well as methylation transferases in KIRC

**FIGURE 4:**
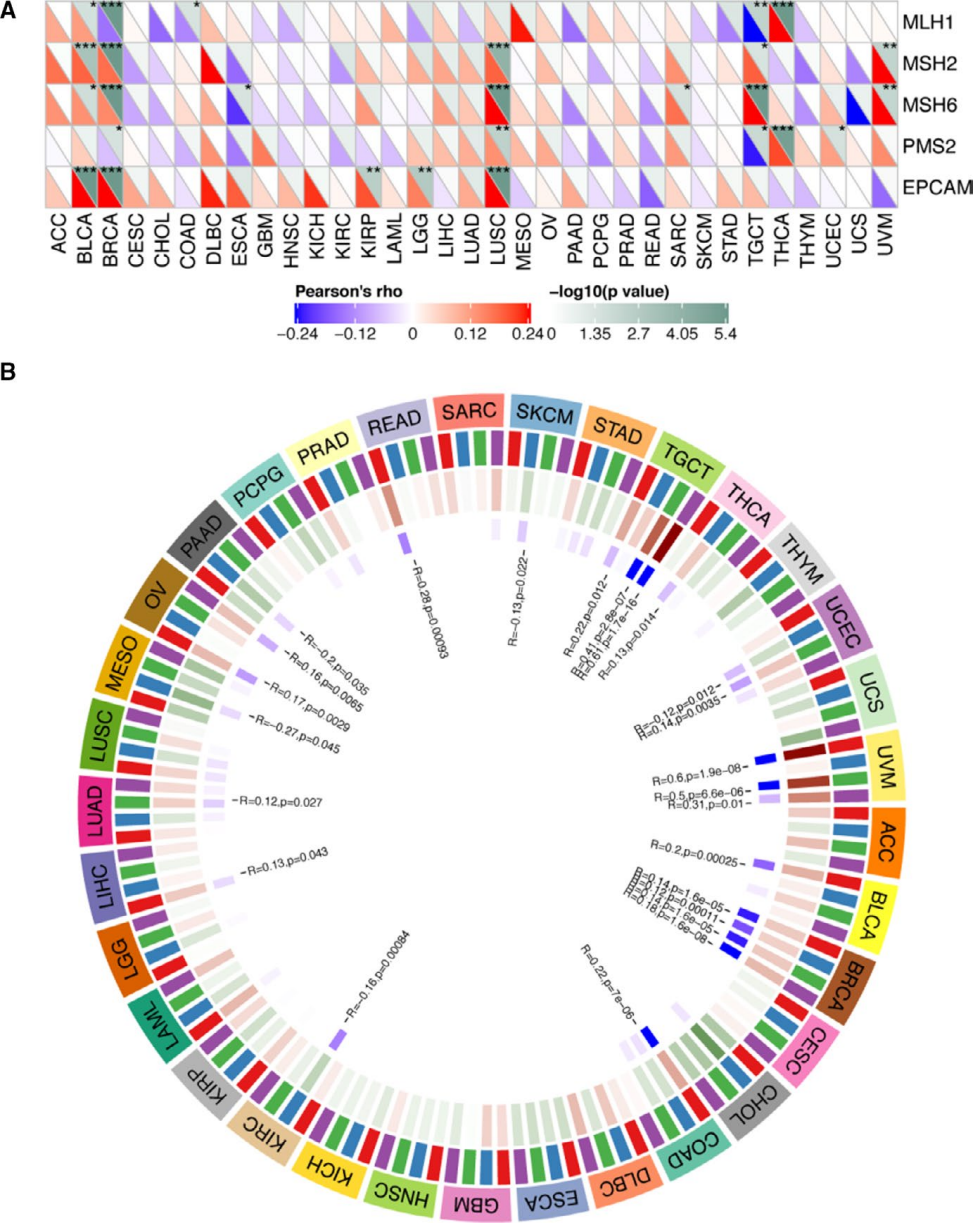
Correlation analyses of CHAC1 and MMR genes as well as methylation transferases in KIRC. A, The correlation analysis of CHAC1 expression and MMR genes. B, The correlation analysis of CHAC1 expression and methylation transferases. (*P < .05; **P < .01; ***P < .001)

To investigate the link between CHAC1 expression and tumorigenesis mechanisms, we examined the relationship between CHAC1 and acknowledged mismatch repair genes like MutL Homolog 1 (MLH1), MutS Homolog 2 (MSH2), MutS Homolog 6 (MSH6), PMS1 Homolog 2, Mismatch Repair System Component (PMS2) and Epithelial Cell Adhesion Molecule (EPCAM). However, no statistic relationship was noticed between the expression of CHAC1 and these MMR genes in KIRC (Figure 4A). We further investigated the possible correlation of CHAC1 and four methylation transferase genes such as DNA Methyltransferase 1 (DNMT1), DNA Methyltransferase 2 (DNMT2), DNA Methyltransferase 3 Alpha (DNMT3A) and DNA Methyltransferase 3 Beta (DNMT3B). The result revealed that the expression of CHAC1 is associated with DNMT2 with a *P* value of .00084 in KIRC (Figure 4B).

### 3.5 Gene set enrichment analysis of CHAC1 in KIRC

**FIGURE 5:**
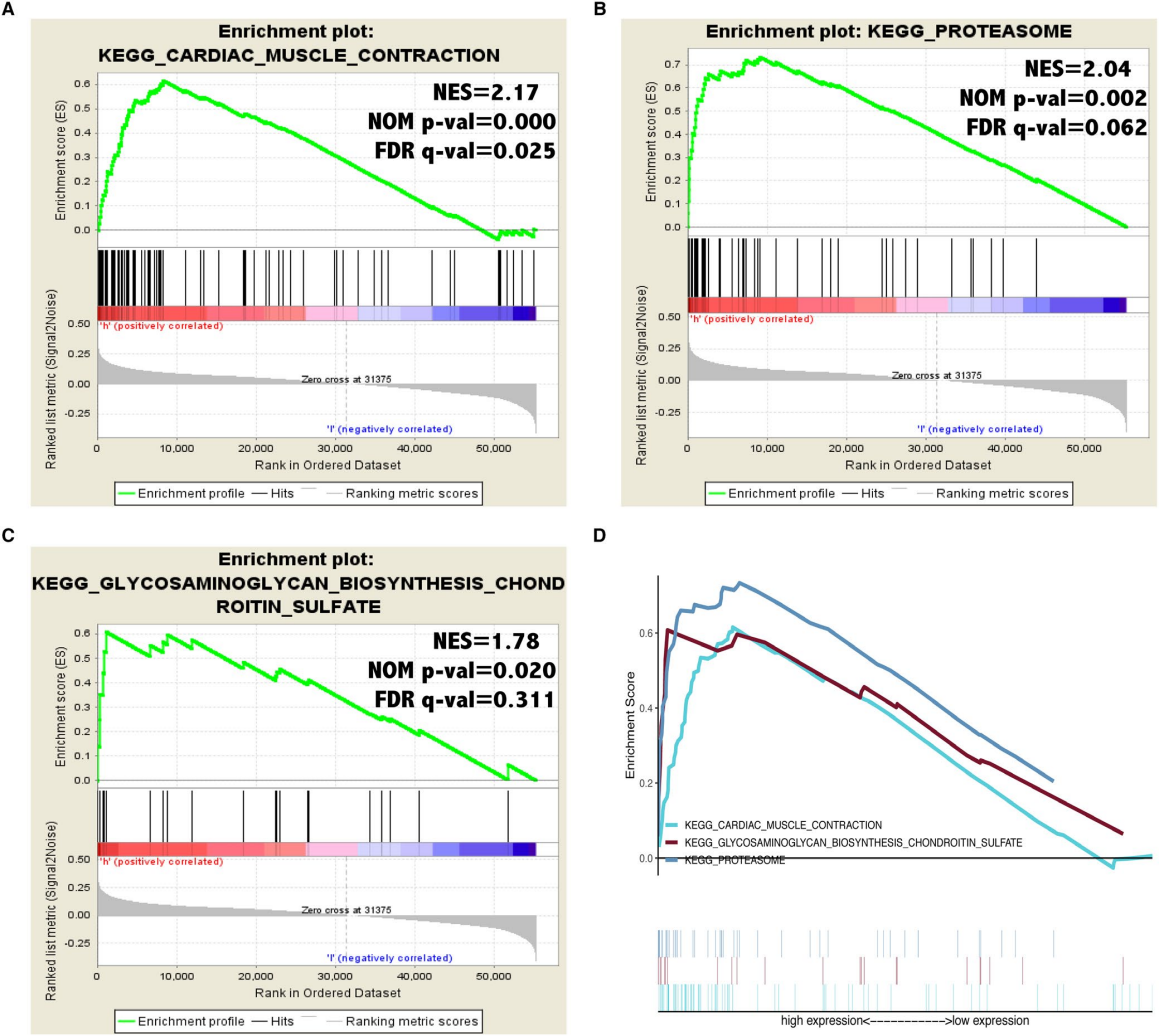
Gene Set Enrichment Analysis of CHAC1 in KIRC. A, 'cardiac muscle contraction'. B, 'proteasome'. C, 'glycosaminoglycan biosynthesis chondroitin sulfate'. D, Summarizing of three signalling pathways

We used the Gene Set Enrichment Analysis to identify potential signaling pathways might be activated or inhibited because of the differential expression of CHAC1 in KIRC. And we selected three most significantly enriched signaling pathways based on the normalized enrichment score (NES) > 1 and Nominal *P*‐value <.05, namely the cardiac muscle contraction (NSE = 2.17, NOM *P*‐value = .000), proteasome (NSE = 2.04, NOM *P*‐value = .002) and glycosaminoglycan biosynthesis chondroitin sulfate (NSE = 1.78, NOM *P*‐value = .020) (Figure 5A‐D).

### 3.6 Evaluate the function of CHAC1 in vitro

**FIGURE 6:**
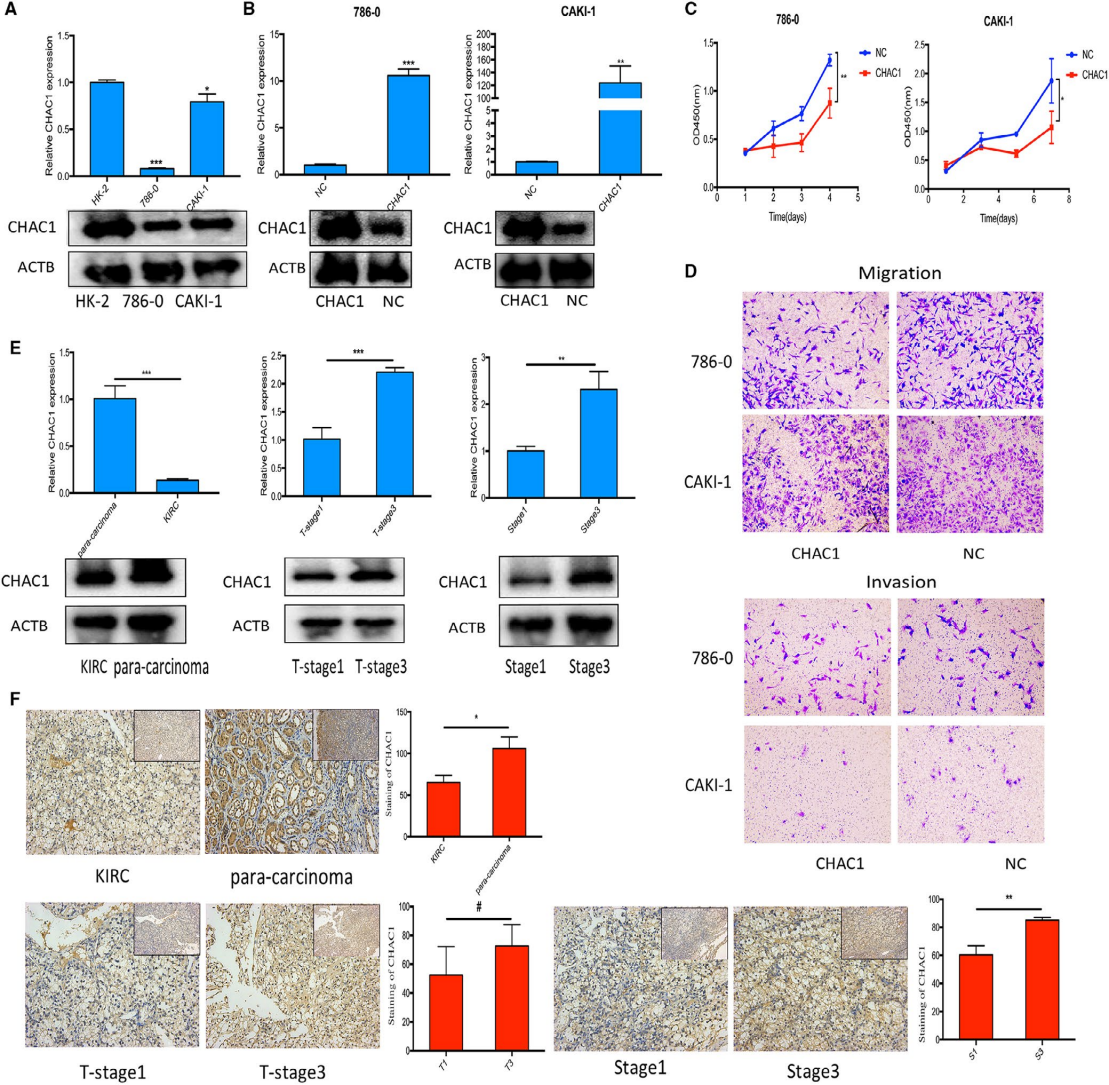
Evaluate the function of CHAC1 in vitro. A, The qRT‐PCR and western blot of CHAC1 mRNA and protein expression in KIRC cell lines 786‐0 and CAKI‐1 and renal tubular epithelial cell HK‐2. B, The qRT‐PCR and western blot of CHAC1 mRNA and protein expression in KIRC cell lines 786‐0 and CAKI‐1 transfected with CHAC1 overexpression vector or negative control vector. C, CCK‐8 experiments of 786‐0 and CAKI‐1 transfected with CHAC1 overexpression vector or negative control vector. D, Cell migration and transwell experiments of 786‐0 and CAKI‐1 transfected with CHAC1 overexpression vector and negative control vector. E, The qRT‐PCR and western blot of CHAC1 mRNA and protein expression in KIRC samples and pericarcinous tissues extracted from patients who underwent surgical treatment for KIRC. F, Immunohistochemistry of CHAC1 in KIRC samples and pericarcinous tissues extracted from patients who underwent surgical treatment for KIRC. (^#^ *P* > .05; *P < .05; **P < .01; ***P < .001)

By qRT‐PCR and western blot, we confirmed the mRNA and protein expression of CHAC1 is down‐regulated in KIRC cell lines 786‐0 and CAKI‐1 when compared with renal tubular epithelial cell HK‐2 (Figure 6A). Furthermore, we investigated the mRNA and protein expression of CHAC1 using qRT‐PCR, western blot and immunohistochemistry in KIRC samples and the pericarcinous tissues collected in our institution. The results of qRT‐PCR and western blot revealed the mRNA and protein expression of CHAC1 is down‐regulated in KIRC tissues when compared with corresponding pericarcinous tissues. However, among these tumour samples with different T stages and total stages, we found KIRC samples extracted from higher T stage or higher total stage usually exhibit relatively higher CHAC1 mRNA or protein expression (Figure 6E). The result from immunohistochemistry was similar with the results of qRT‐PCR and western blot though no statistic difference about the staining of CHAC1 was noticed between the KIRC samples with T1 stage and T3 stage (Figure 6F). CHAC1 overexpression vector was used to investigate the function of CHAC1 in KIRC cell lines. The transfection efficiency was verified using qRT‐PCR and western blot (Figure 6B). The results of CCK‐8 revealed that over‐expression of CHAC1 significantly induced cell death (Figure 6C). However, the results from cell migration and invasion assays showed over‐expression of CHAC1 had limited influence on cell migration and invasion in KIRC cell lines (Figure 6D).

## 4 DISCUSSION

Kidney renal clear cell carcinoma was found featured with the GSH metabolism abnormalities and highly sensitive to the depletion of GSH.^20^ It has been proved that the depletion of GSH is associated with the ROS accumulation and ferroptosis activation.^8^, ^9^ CHAC1 is a newly discovered endoplasmic reticulum inducible gene,^21^ involved in the γ‐glutamyl cycle that can degrade glutathione^22^, ^23^ and promote cell apoptosis or ferroptosis.^24^, ^25^ Previous research has also demonstrated that CHAC1 is differentially expressed in KIRC.^11^ However, to our knowledge, there have been no researches investigating the value of CHAC1 in the prognosis or therapeutic potential in KIRC.

By differential genes expression analysis, we found CHAC1 is down‐regulated in KIRC samples when compared with normal kidney samples. But when we compared the expression of CHAC1 in KIRC samples with different grades and stages, we noticed CHAC1 is up‐regulated in relatively higher malignancy or later stage of KIRC. These controversial findings obfuscate the role of CHAC1 in the initiation or the progress of KIRC. By reviewing existing literature, we noticed elevated expression of CHAC1 or its splicing variants could predict poor outcomes in uveal melanoma patients^26^ or in breast and ovarian cancer patients^27^ respectively. To explore the potential role of CHAC1 in KIRC, we conducted survival analysis as well as the univariate and multivariate cox regression analysis using the sequencing data and clinical information derived from TCGA database. And the results revealed the up‐regulated CHAC1 could predict the poor prognosis of KIRC. Besides, we also investigated the expression of CHAC1 in KIRC samples and pericarcinous tissues collected in our institution. The results revealed CHAC1 is down‐regulated in KIRC tissues when compared with corresponding pericarcinous tissues, but up‐regulated in KIRC samples with relatively higher total stage. These results are similar with the results from previous researches as we mentioned above. Furthermore, we also transfected KIRC cell lines 786‐0 and CAKI‐1 with CHAC1 overexpression vector or it negative control vector to investigate the function of CHAC1 in KIRC cell lines. And the results revealed overexpression of CHAC1 significantly inhibited cell proliferation and induced cell death, but cast limited influence on the abilities of cell migration and invasion. It is reported dysregulated GSH metabolism widely exist in malignant tumours.^28^, ^29^, ^30^ And Heike Miess has demonstrated that higher levels of GSH were detected in higher malignancy and later stage of KIRC.^10^ As a result, we speculated the up‐regulated CHAC1 in relatively higher malignancy or later stage of KIRC noticed in our research might be the feedback of high levels of GSH and further researches are still needed to reveal the function of CHAC1 in KIRC.

To explore the potential of CHAC1 for the immunotherapy of KIRC, correlation analyses were conducted and no clear association between CHAC1 and immunotherapy related features such as neoantigens, MSI, TMB and tumour microenvironment was noticed. However, the correlation analysis of CHAC1 and immune cell related immune pathway revealed differential expression of CHAC1 may be associated with memory B cell, natural killer cell and type1 T helper cell related immune pathway. The correlation analysis of CHAC1 and checkpoint genes revealed differential expression of CHAC1 may be associated with checkpoint genes like ADORA2A, CD200, CD44, CD70, HHLA2, NRP1, PDCD1LG2 and TNFRSF18. To the best of our knowledge, there have been no researches concerning the role of memory B cell, type1 T helper cell as well as the checkpoint genes like ADORA2A and TNFRSF18 in the immunotherapy of KIRC, let alone the association between CHAC1 and these immunologically relevant targets. These results may provide new sights for the potential of immunotherapy for KIRC. Besides, we noticed the expression of CHAC1 is associated with various gene markers of immune pathway as well as the checkpoint genes in LUAD, LUSC, PRAD, BRAC and especially, in UVM. The results indicated the differential expression of CHAC1 may play an important role in the immunotherapy of these cancers.

We further studied the role of CHAC1 in tumorigenesis by investigating the possible correlation between CHAC1 and MMR genes as well as methylation transferases. And the results showed CHAC1 is associated with the methylation transferase DNMT2. To our knowledge, the correlation between these two genes has been barely studied. Gene Set Enrichment Analysis was also conducted and the result indicated CHAC1 may influence KIRC progression through the pathway of the cardiac muscle contraction, proteasome and glycosaminoglycan biosynthesis chondroitin sulfate. Although no researches on these correlation have been conducted.

To sum up, in current research, we found CHAC1 is down‐regulated in KIRC samples when compared with normal samples. However, among KIRC samples with different grades and stages, CHAC1 is up‐regulated in relatively higher malignancy and later stage of KIRC. The survival curve and multivariate cox regression analysis indicated up‐regulated CHAC1 is an independent risk factor for poor prognosis of KIRC. A nomogram model based on the result of multivariate cox regression analysis was constructed and effectively predicted patients survival rate.

## CONFLICT OF INTEREST

The authors declare that they have no conflict of interest.

## AUTHOR CONTRIBUTIONS

**Deng Li:** Data curation (equal); Formal analysis (equal); Investigation (equal); Methodology (equal). **Shiwei Liu:** Data curation (equal); Formal analysis (equal); Visualization (equal). **Jie Xu:** Data curation (equal); Formal analysis (equal); Writing‐original draft (equal). **Lei Chen:** Visualization (equal); Writing‐review & editing (equal). **Chaoliang Xu:** Data curation (equal); Formal analysis (equal); Writing‐review & editing (equal). **Feiteng Chen:** Data curation (equal); Formal analysis (equal); Writing‐review & editing (equal). **Zijie Xu:** Data curation (equal); Formal analysis (equal). **Yu Zhang:** Data curation (equal); Formal analysis (equal); Writing‐review & editing (equal). **shujie xia:** Conceptualization (equal); Methodology (equal); Project administration (equal); Supervision (equal). **Yi Shao:** Conceptualization (equal); Methodology (equal); Project administration (equal); Writing‐review & editing (equal). **Yi Wang:** Conceptualization (equal); Methodology (equal); Project administration (equal).

## Supporting information

Fig S1
