# Corrigendum

**DOI:** 10.1111/jcmm.17506

**Published:** 2022-09-11

In Deng Li et al.^1^ the published article contains error in Figure 3G. The correct figure is shown below. The authors confirm all results, and conclusions of this article remain unchanged.

**FIGURE 3:**
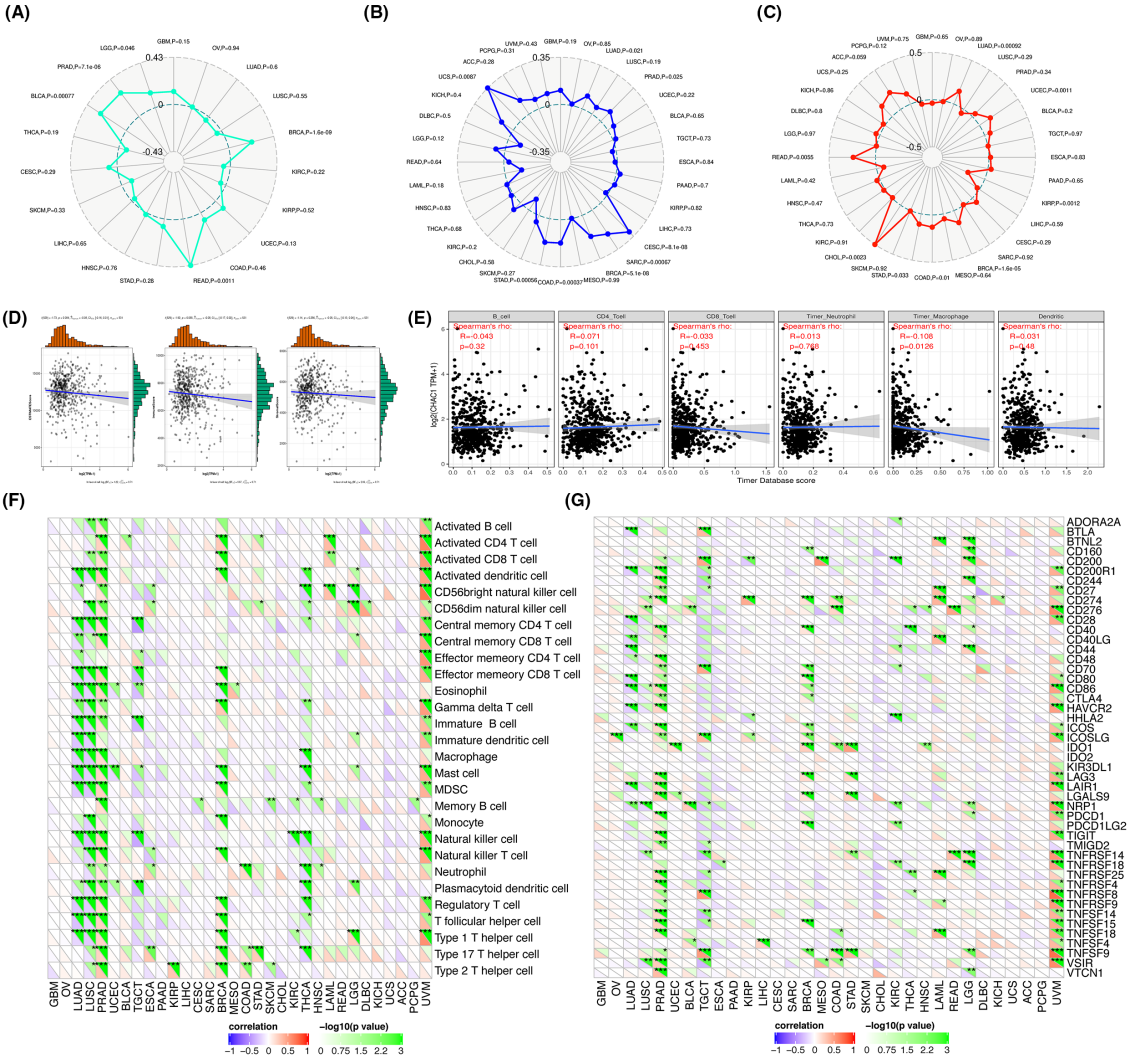
Immunological features related to CHAC1 in KIRC. The correlation analysis of CHAC1 expression and neoantigens. (B) The correlation analysis of CHAC1 expression and MSI. (C) The correlation analysis of CHAC1 expression and TMB. (D) ESTIMATE: The correlation analysis of CHAC1 expression and tumour microenvironment. (E) TIMER: The correlation analysis of CHAC1 expression and infiltration of immune cells. (F) The correlation analysis of CHAC1 expression and acknowledged markers of immune cells. (G) The correlation analysis of CHAC1 expression and checkpoint genes. (*p < 0.05; **p < 0.01; ***p < 0.001).
