# Supplementary material for: Ferroptosis‐related gene CHAC1 is a valid indicator for the poor prognosis of kidney renal clear cell carcinoma
Source: J Cell Mol Med. 2021 Mar 16;25(7):3610–21. doi: 10.1111/jcmm.16458 (PMC8034464; doi:10.1111/jcmm.16458)
Supplement: Supplementary file 1 — Fig S1 [file JCMM-25-3610-s001.doc]

**Supplemental Figure 1**


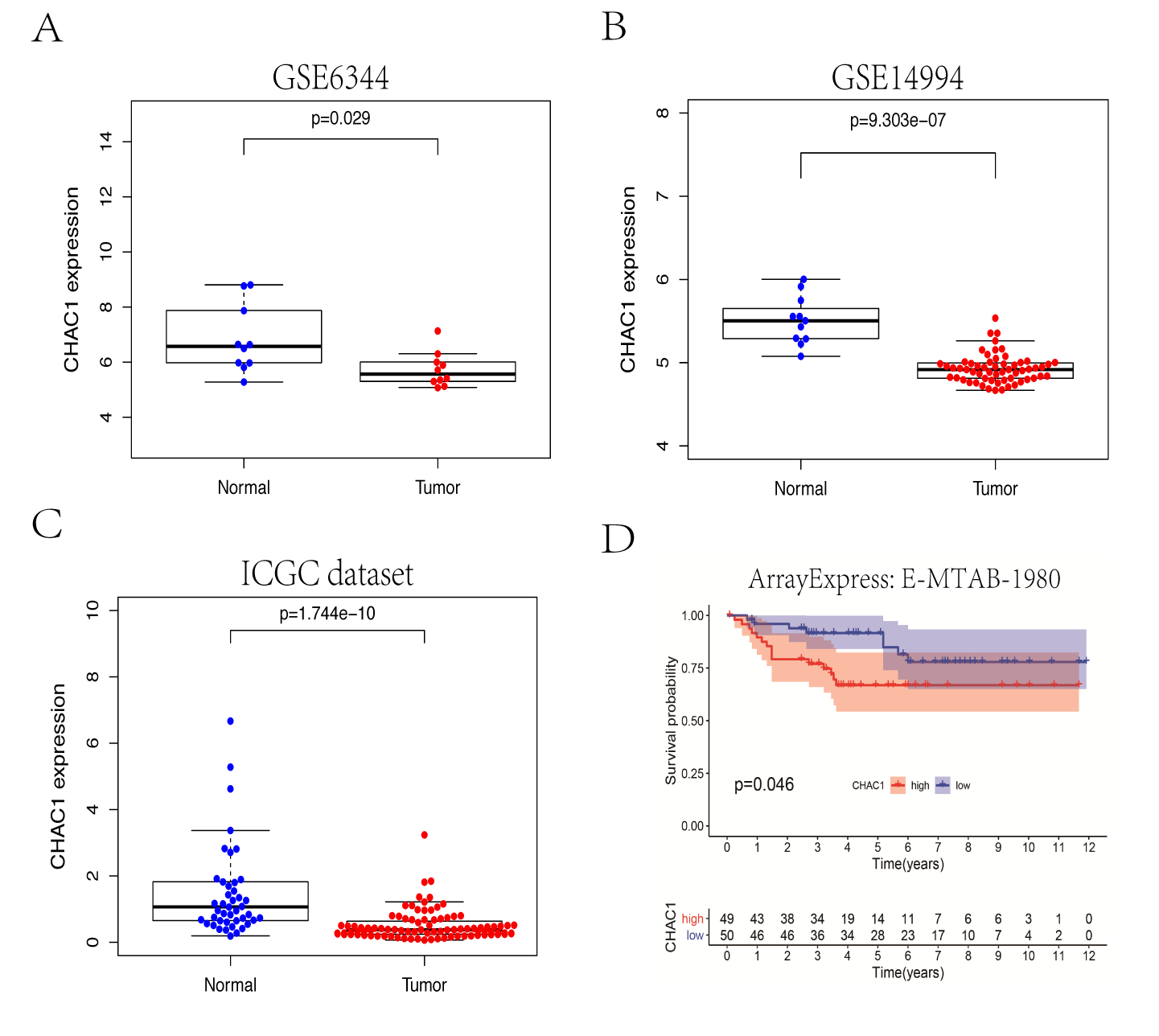


**Supplemental Figure 1. External verification of the differential expression and predict value of CHAC1 in KIRC**

(A) The differential expression of CHAC1 in GEO dataset of GSE6344. (B) The differential expression of CHAC1 in GEO dataset of GSE14994. (C) The differential expression of CHAC1 in ICGC dataset. (D) The overall survival curve of KIRC in ArrayExpress dataset of E-MTAB-1980.
